# Sex Differences in Risk Profile, Stroke Cause and Outcome in Ischemic Stroke Patients With and Without Migraine

**DOI:** 10.3389/fnins.2021.740639

**Published:** 2021-11-03

**Authors:** Katie M. Linstra, Hendrikus J. A. van Os, Ynte M. Ruigrok, Paul J. Nederkoorn, Ewoud J. van Dijk, L. Jaap Kappelle, Peter J. Koudstaal, Marieke C. Visser, Michel D. Ferrari, Antoinette MaassenVanDenBrink, Gisela M. Terwindt, Marieke J. H. Wermer

**Affiliations:** ^1^Department of Neurology, Leiden University Medical Center, Leiden, Netherlands; ^2^Division of Pharmacology and Vascular Medicine, Department of Internal Medicine, Erasmus MC, Rotterdam, Netherlands; ^3^Department of Neurology and Neurosurgery, UMC Utrecht Brain Center, University Medical Center Utrecht, Utrecht, Netherlands; ^4^Department of Neurology, Amsterdam University Medical Center, Amsterdam, Netherlands; ^5^Department of Neurology, Radboud University Medical Center, Nijmegen, Netherlands; ^6^Department of Neurology, Erasmus Medical Center, Rotterdam, Netherlands

**Keywords:** sex differences, migraine, stroke outcome, stroke subtype, cardiovascular risk factors

## Abstract

**Background:** An increased risk of stroke in patients with migraine has been primarily found for women. The sex-dependent mechanisms underlying the migraine–stroke association, however, remain unknown. This study aims to explore these sex differences to improve our understanding of pathophysiological mechanisms behind the migraine–stroke association.

**Methods:** We included 2,492 patients with ischemic stroke from the prospective multicenter Dutch Parelsnoer Institute Initiative study, 425 (17%) of whom had a history of migraine. Cardiovascular risk profile, stroke cause (TOAST classification), and outcome [modified Rankin scale (mRS) at 3 months] were compared with both sexes between patients with and without migraine.

**Results:** A history of migraine was not associated with sex differences in the prevalence of conventional cardiovascular risk factors. Women with migraine had an increased risk of stroke at young age (onset < 50 years) compared with women without migraine (RR: 1.7; 95% CI: 1.3–2.3). Men with migraine tended to have more often stroke in the TOAST category other determined etiology (RR: 1.7; 95% CI: 1.0–2.7) in comparison with men without migraine, whereas this increase was not found in women with migraine. Stroke outcome was similar for women with or without migraine (mRS ≥ 3 RR 1.1; 95% CI 0.7–1.5), whereas men seemed to have a higher risk of poor outcome compared with their counterparts without migraine (mRS ≥ 3 RR: 1.5; 95% CI: 1.0–2.1).

**Conclusion:** Our results indicate possible sex differences in the pathophysiology underlying the migraine–stroke association, which are unrelated to conventional cardiovascular risk factors. Further research in larger cohorts is needed to validate these findings.

## Introduction

Migraine is a prevalent brain disorder and important risk factor for cardiovascular disease (CVD), including stroke. The increased risk is especially evident in women and less clear in men (Schurks et al., 2009). In addition, sex differences in ischemic stroke are increasingly acknowledged. Women more often suffer from ischemic stroke compared with men, especially after menopause, and have an increased risk of poor outcome (Gall et al., 2012; Bushnell et al., 2014). Although it has been recognized that cardiovascular pathophysiology is partly different between women and men, the role of sex in the migraine–stroke association remains poorly understood (Reeves et al., 2008; Schurks et al., 2009; Haast et al., 2012). Missing gaps in the association are the role of conventional and non-conventional vascular risk factors, the relation with underlying stroke cause, and the effect of migraine susceptibility on brain tissue recovery after ischemia. Until now, it is unknown how sex affects these factors.

This explorative study aims to investigate differences in cardiovascular risk profiles, stroke cause, and stroke outcome between men and women to improve our understanding of pathophysiological mechanisms underlying the migraine–stroke association.

## Materials and Methods

We selected patients with ischemic stroke for whom information on a history of migraine was available from the prospective registry and biobank “Dutch Parelsnoer Institute Cerebrovascular Accident (PSI-CVA) Initiative” in eight university hospitals in the Netherlands (Nederkoorn et al., 2015). The PSI-CVA registry is a large cohort of stroke patients in which comprehensive clinical data, detailed phenotyping of stroke, imaging data, and biomaterials were prospectively and uniformly collected. The registry started in 2009 and ended in 2019. The Ethics Committees of all participating centers approved the PSI-CVA Initiative.

Data on cardiovascular risk profile (conventional risk factors including smoking, diabetes mellitus, hyperlipidemia, previous stroke, myocardial infarction, atrial fibrillation, BMI ≥ 25, and hypertension) and stroke classification were obtained prospectively upon hospital admission. Ischemic stroke was defined according to the WHO criteria and confirmed on CT or MRI and further specified according to the trial of ORG 10172 in acute stroke treatment (TOAST) classification in the subcategories large-artery atherosclerosis, cardioembolism, small-vessel occlusion, stroke of other determined etiology, and stroke of undetermined etiology (Adams et al., 1993). The modified Rankin Scale (mRS) was used to grade stroke outcome. A poor outcome was defined as mRS at 3 months after discharge ≥ 3.

Migraine history was prospectively obtained at hospital admission using a short, validated questionnaire that was specially developed to establish migraine diagnosis in patients with stroke (MISS questionnaire, see Supplementary Material; van der Willik et al., 2016).

We performed a complete case analysis with respect to migraine status. Poisson regression analysis was performed to calculate risk ratios (RR) including 95% confidence intervals (CI) for the associations between age of stroke onset, cardiovascular risk factors, stroke subtype and outcome, and migraine diagnosis, for all patients and for each sex separately. The analyses were adjusted for potential confounders.

## Results

In total 6,259 participants were included in the PSI-CVA database, of whom 4,273 had ischemic stroke and 2,492 (40% women) also with information on migraine status. A lifetime history of migraine was present in 425/2,492 (17% overall, 10% in men, and 27% in women) of the participants. Age, sex, and cardiovascular risk profile were similar between patients with or without available information about migraine status.

There were no differences in cardiovascular risk factor profile in stroke patients with vs. without migraine overall or between sexes (Table 1).

**TABLE 1:** Demographics and cardiovascular risk factors.

	All				Women				Men			
	Migraine	No migraine	RR	aRR^a^	Migraine	No migraine	RR	aRR^b^	Migraine	No migraine	RR	aRR^b^
**Demographics**												
Number	425 (1)	2,067 (83)	–	–	264 (62)	730 (35)	–	–	161 (38)	1,337 (65)	–	–
Age, years	61 ± 15	67 ± 14*	–	–	61 ± 17	68 ± 15*	–	–	61 ± 13	66 ± 14*	–	–
Age of onset < 50	93 (22)	266 (13)	1.7 (1.3–2.1)	–	65 (25)	105 (14)	1.7 (1.3–2.3)	–	28 (17)	161 (12)	1.4 (0.9–2.1)	–
Age of onset ≥ 50	332 (78)	1,801 (87)	0.8 (0.8–0.9)	–	199 (75)	625 (86)	0.9 (0.7–1.0)	–	133 (83)	1,176 (88)	0.9 (0.8–1.1)	–
Pre-stroke mRS	29 (7)	164 (9)	0.8 (0.5–1.2)	0.9 (0.6–1.3)	21 (8)	82 (12)	0.7 (0.4–1.1)	0.8 (0.5–1.3)	8 (5)	82 (7)	0.8 (0.4–1.5)	0.9 (0.4–1.8)
**CV risk factors**												
Hypertension^c^	226 (54)	1,115 (54)	1.0 (0.9–1.1)	1.1 (1.0–1.3)	138 (53)	412 (57)	0.9 (0.8–1.1)	1.1 (0.9–1.3)	88 (55)	703 (53)	1.0 (0.8–1.3)	1.1 (0.9–1.4)
DM^d^	58 (14)	304 (15)	0.9 (0.7–1.2)	1.1 (0.8–1.4)	35 (13)	113 (16)	0.9 (0.6–1.2)	1.0 (0.7–1.5)	23 (14)	191 (14)	1.0 (0.6–1.5)	1.1 (0.7–1.7)
Hyperlipidemia^e^	145 (35)	752 (37)	0.9 (0.8–1.1)	1.1 (0.9–1.3)	89 (34)	240 (34)	1.0 (0.8–1.3)	1.2 (0.9–1.5)	56 (35)	512 (39)	0.9 (0.7–1.2)	1.0 (0.7–1.3)
Previous Stroke	115 (28)	515 (26)	1.1 (0.9–1.3)	1.2 (1.0–1.5)	69 (27)	169 (24)	1.1 (0.9–1.5)	1.3 (1.0–1.7)	46 (29)	346 (27)	1.1 (0.8–1.5)	1.2 (0.9–1.6)
History of MI	34 (8)	262 (13)	0.6 (0.4–0.9)	0.9 (0.6–1.3)	15 (6)	57 (8)	0.7 (0.4–1.3)	0.9 (0.5–1.6)	19 (12)	205 (16)	0.8 (0.5–1.2)	1.0 (0.6–1.5)
Atrial fibrillation	42 (10)	259 (13)	0.8 (0.6–1.1)	1.1 (0.8–1.6)	22 (9)	81 (11)	0.8 (0.5–1.2)	1.0 (0.6–1.7)	20 (13)	178 (14)	0.9 (0.6–1.5)	1.2 (0.8–2.0)
Smoking ever^f^	25 (6)	186 (9)	0.7 (0.4–1.0)	0.8 (0.5–1.2)	15 (6)	50 (7)	0.8 (0.5–1.4)	0.9 (0.5–1.7)	10 (6)	136 (10)	0.6 (0.3–1.1)	0.7 (0.3–1.3)
BMI ≥ 25	258 (62)	1,267 (64)	1.0 (0.8–1.1)	1.1 (0.9–1.2)	147 (57)	349 (51)	1.1 (0.9–1.4)	1.1 (0.9–1.3)	111 (69)	918 (71)	1.0 (0.8–1.2)	1.0 (0.8–1.2)

*mRS, modified Rankin Scale; CV, cardiovascular; DM, diabetes mellitus; MI, myocardial infarction; BMI, body mass index (kg/m^2^).*

*Data are represented as mean ± SD or number of subjects (%).*

*Migraine vs. no migraine: p < 0.001.

*^*a*^Adjusted for age and sex.*

*^*b*^Adjusted for age.*

*^*c*^Ever or current diagnosis or treatment with antihypertensive drugs.*

*^*d*^Ever or current diagnosis or treatment with antidiabetic drugs.*

*^*e*^Total cholesterol > 3.5 mmol/L, low-density lipoprotein cholesterol > 2.5 mmol/L or treatment with lipid-lowering agents.*

*^*f*^Current smokers and smokers who stopped smoking > 6 months ago.*

**FIGURE 1:**
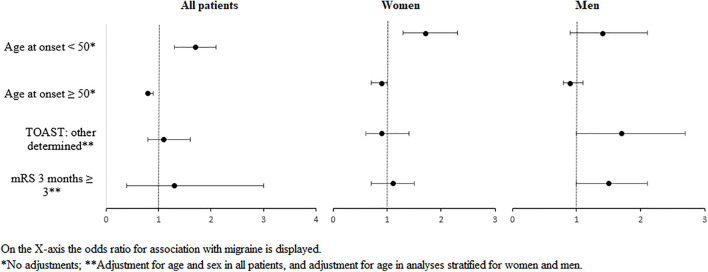
Forest plot of the most important findings on associations between migraine and risk factors, etiology, or outcome of stroke, stratified for sex.

Women with migraine had their stroke on average 7 years (*p* < 0.0001) and men 5 years earlier than stroke patients without migraine (*p* < 0.0001). Stroke onset < 50 years occurred more often in women with than in women without migraine (RR: 1.7; 95% CI: 1.3–2.3, Table 1 and Figure 1). This increased risk could not be confirmed in men (RR: 1.4; 95% CI: 0.9–2.1).

Men with migraine tended to have a higher risk for stroke of other determined etiology compared with men without migraine (RR: 1.7; 95% CI: 1.0–2.7), whereas no differences in this TOAST category were found in women (RR: 0.9; 95% CI: 0.6–1.4, Table 2 and Figure 1). Other stroke subtypes were comparable with men and women with and without migraine, although the effect estimate had an opposite direction for the category small vessel occlusion.

**TABLE 2:** Stroke subtype according to TOAST classification.

	All				Women				Men			
	Migraine	No migraine	RR	RR^a^	Migraine	No migraine	RR	RR^b^	Migraine	No migraine	RR	RR^b^
LAA	84 (20)	530 (26)	0.8 (0.6–1.0)	0.9 (0.7–1.2)	45 (17)	147 (21)	0.8 (0.6–1.2)	0.9 (0.7–1.3)	39 (24)	383 (29)	0.8 (0.6–1.1)	0.9 (0.7–1.3)
Cardioembolism	50 (12)	296 (15)	0.8 (0.6–1.1)	0.9 (0.7–1.2)	32 (12)	102 (14)	0.7 (0.6–1.3)	1.0 (0.7–1.5)	18 (11)	194 (15)	0.8 (0.5–1.2)	0.8 (0.5–1.3)
SVO	72 (17)	373 (18)	0.9 (0.7–1.2)	0.9 (0.7–1.2)	43 (17)	158 (22)	0.8 (0.5–1.0)	0.8 (0.6–1.1)	29 (18)	215 (16)	1.1 (0.7–1.6)	1.1 (0.7–1.6)
Other determined	47 (11)	137 (7)	1.7 (1.2–2.3)	1.1 (0.8–1.6)	27 (10)	59 (8)	1.3 (0.8–2.0)	0.9 (0.6–1.4)	20 (12)	78 (6)	2.1 (1.3–3.4)	1.7 (1.0–2.7)
Undetermined	167 (40)	692 (34)	1.2 (1.0–1.4)	1.1 (0.9–1.3)	113 (43)	250 (35)	1.2 (1.0–1.6)	1.2 (0.9–1.5)	54 (34)	442 (34)	1.0 (0.8–1.3)	1.0 (0.7–1.3)

*TOAST, Trial of ORG 10172 in acute stroke treatment; LAA, large-artery atherosclerosis; SVO, small-vessel occlusion.*

*Data are represented as mean ± SD or number of subjects (%).*

*^*a*^Adjusted for age and sex.*

*^*b*^Adjusted for age.*

Outcome after stroke seemed to be comparable with women regardless of migraine diagnosis (RR: 1.1; 95% CI: 0.7–1.5), whereas men tended to have a worse outcome compared with their counterparts without migraine (RR: 1.5; 95% CI: 1.0–2.1, Table 3 and Figure 1).

**TABLE 3:** Stroke severity and outcome.

	All				Women				Men			
	Migraine	No migraine	RR	RR^a^	Migraine	No migraine	RR	RR^b^	Migraine	No Migraine	RR	RR^b^
NIHSS ≥ 7^C^	67 (17)	340 (18)	0.9 (0.7–1.2)	0.9 (0.7–1.2)	36 (15)	131 (20)	0.7 (0.5–1.1)	0.8 (0.5–1.1)	31 (21)	209 (17)	1.2 (0.8–1.1)	1.2 (0.8–1.8)
mRS discharge ≥ 3	102 (30)	238 (32)	1.0 (0.8–1.2)	1.1 (0.9–1.4)	59 (28)	184 (32)	0.9 (0.7–1.2)	1.2 (0.8–1.6)	43 (33)	307 (32)	1.1 (0.8–1.4)	1.2 (0.8–1.6)
mRS 3 months ≥ 3	73 (20)	368 (20)	1.0 (0.7–1.2)	1.3 (0.9–1.7)	41 (18)	142 (22)	0.8 (0.6–1.1)	1.1 (0.7–1.5)	32 (23)	226 (19)	1.2 (0.8–1.7)	1.5 (1.0–2.1)

*NIHSS, National Institute of Health Stroke Scale; mRS, modified Rankin Scale.*

*Data are represented as mean ± SD or number of subjects (%).*

*^*a*^Adjusted for pre-stroke mRS, NIHSS at admission (for mRS at discharge and at 3 months), age, and sex.*

*^*b*^Adjusted for pre-stroke mRS, NIHSS at admission (for mRS at discharge and at 3 months) and age.*

*^*c*^NIHSS on admission.*

## Discussion

Our explorative study suggests that sex differences in stroke pathophysiology in patients with migraine cannot be explained by differences in conventional vascular risk factors. Women with migraine had a higher risk for stroke under the age of 50. Men tended to more often have stroke of other determined etiology and a worse outcome compared with men without migraine.

Evidence in the literature about the relationship between conventional vascular risk factors and migraine is conflicting, and rarely, data of men and women are analyzed separately (Sacco et al., 2015). In general, the association between migraine and stroke is thought to be more prominent in patients without a traditional vascular risk profile and with a lower Framingham Risk Score (Li et al., 2015; Sacco et al., 2015). Only little is known about the association between migraine and sex-specific cardiovascular risk factors. Unfortunately, our PSI-CVA database did not contain all factors needed to construct Framingham Risk score. Also, our database did not include non-conventional sex-dependent vascular risk factors such as (pre)-eclampsia, sex hormone disorders, or use of hormones. Future studies are therefore needed to investigate the effect of these non-conventional risk factors. A younger age at stroke onset in patients with migraine in general, has been reported previously (Schurks et al., 2009; Li et al., 2015).

Previous studies on stroke etiology reported lower frequencies of large vessel and cardio-embolic stroke etiology in female migraine patients and more infarcts of unknown origin in migraine patients in general (Rist et al., 2010; Li et al., 2015). In a recent study, migraine with aura was strongly associated with cryptogenic stroke, whereas such association was not found in migraine without aura (Martinez-Majander et al., 2021). The association of migraine with aura with stroke was independent of vascular risk factors or patent foramen ovale. The association was present in both women and men, although the odds ratios were higher in women. We observed an increase in stroke of other determined etiology only in men with migraine (with and without aura combined). Sex differences in migraine pathophysiology are likely multifactorial and may reflect genetic and hormonal sex differences. In addition, migraine is associated with cerebral hyperexcitability and spreading depolarization (SD), the neurophysiological correlate of migraine aura. SD is associated with neurovascular uncoupling and can also be found in the penumbra of cerebral ischemia (Ferrari et al., 2015). These mechanisms may be associated with a sex-specific systemic vascular pathology in migraine patients (Sacco et al., 2015). Since the increased stroke risk in migraine patients is not associated with enhanced atherosclerosis, alternative pathology, including micro-embolisms, vasospasms in the microvasculature and endothelial dysfunction, may be involved (Tietjen, 2009; Stam et al., 2013; Ferrari et al., 2015; van Os et al., 2017). These “non-conventional” mechanisms may explain the higher proportion of other determined causes in men with migraine. We have no good explanation why the higher risk was only found in men and not in women with migraine.

Existing literature on functional stroke outcome in patients with migraine is limited to the Women’s Health Study, which only included female health care employees and reported a relatively favorable mRS at hospital discharge after ischemic stroke for women with migraine with aura (Sacco et al., 2015). In general, female sex has been associated with a less favorable stroke outcome in terms of disability and mortality (Reeves et al., 2008; Haast et al., 2012). Our study found no differences in outcome between women with and without migraine but did not investigate women with migraine aura separately. In men with migraine, our data cautiously suggested a worse outcome compared with their counterparts without migraine. As these are the first data on stroke outcome in men specifically, further research is needed to confirm these findings and investigate underlying causes.

Strengths of our study are the relatively large sample size, prospective design, and the use of standardized definitions of cardiovascular risk factor and stroke characteristics. Also, we compared men and women with stroke directly with their counterparts without migraine. Migraine diagnosis was established with a validated questionnaire, and migraine prevalence was as expected for this population. Our study also has limitations. First, the MISS questionnaire has only moderate positive predictive value for aura symptoms. Therefore, we did not distinguish between migraine with and without aura, although the migraine–stroke connection is particularly apparent in migraine with aura. Second, from 4,273 participants with ischemic stroke in our cohort, only 2,492 had complete data on migraine. Not all PSI-CVA study centers participated in our migraine study. We consider this selection to be random and assume that it did not result in selection bias. Third, we did not correct for multiple comparisons. Finally, although our study included almost 2,500 stroke patients, the sample size in several sub-analyses was low, and therefore, our study should be considered explorative and hypothesis generating. To confirm our findings and to study sex differences in migraine with aura patients separately, studies with far, with over 10 thousands of stroke patients will be necessary (because of the relative low prevalence of migraine with aura). Future studies are also needed to study sex-specific non-conventional cardiovascular risk factors and investigate stroke causes in more detail to enable sex-specific prevention of strokes in patients with migraine.

## Data Availability Statement

The datasets presented in this article are not readily available because anonymized data may be requested for the sole purpose of replicating procedures and results presented in the article and only after agreement of the Dutch Parelsnoer Institute Cerebrovascular Accident (PSI-CVA) Initiative committee. Requests to access the datasets should be directed to corresponding author.

## Ethics Statement

The studies involving human participants were reviewed and approved by the ethics committees of all participating medical centres. The patients/participants provided their written informed consent to participate in this study.

## Author Contributions

MW, KL, HO, AM, and GT contributed to conception and design of the study. YR, MW, and KL organized the database extraction. KL and HO performed the statistical analysis. KL wrote the first draft of the manuscript. MW, HO, MF, MV, LK, PK, AM, and GT wrote sections of the manuscript. All authors contributed to manuscript revision, read, and approved the submitted version.

## Conflict of Interest

The authors declare that the research was conducted in the absence of any commercial or financial relationships that could be construed as a potential conflict of interest.

## Publisher’s Note

All claims expressed in this article are solely those of the authors and do not necessarily represent those of their affiliated organizations, or those of the publisher, the editors and the reviewers. Any product that may be evaluated in this article, or claim that may be made by its manufacturer, is not guaranteed or endorsed by the publisher.
