# Supplementary material for: Sex Differences in Risk Profile, Stroke Cause and Outcome in Ischemic Stroke Patients With and Without Migraine
Source: Front Neurosci. 2021 Nov 3;15:740639. doi: 10.3389/fnins.2021.740639 (PMC8597840; doi:10.3389/fnins.2021.740639)
Supplement: Supplementary file 1 [file Data_Sheet_1.PDF]

ONLINE ONLY

SUPPLEMENTARY MATERIAL

Migraine Screener for Stroke (MISS) questionnaire

Migraine Screener for Stroke (MISS) is a 5 question reliable screening tool for migraine diagnosis in patients with transient ischemic attack or stroke. The screener was validated through semi-structured telephone interviews with the International Classification of Headache Disorders, second-edition criteria as gold standard. The sensitivity of all questions combined was 0.47 (95% CI 0.31-0.62), the specificity was 0.97 (95% CI 0.93-0.99), the positive predictive value (PPV) was 0.80 (95% CI 0.59-0.93) and the negative predictive value (NPV) was 0.87 (95% CI 0.82-0.92). For assessing migraine with aura, the question about visual disturbances had a good NPV (0.99, 95% CI 0.96-1.00), but a low PPV (0.38, 95% CI 0.24-0.53). To prevent misclassification, especially for the aura symptoms, patients with a positive screener should be interviewed more extensively to confirm the migraine diagnosis.[1]

Question 1. Have you ever had a migraine attack or do you still have migraine attacks?<br>Never<br>1–4 times<br>≥5 times<br>Unknown
Question 2. Have you ever been diagnosed with migraine by a physician?<br>Yes<br>No<br>Unknown
Question 3. Have you ever had severe headache attacks accompanied by nausea or vomiting?<br>Never<br>1–4 times<br>≥5 times<br>Unknown
Question 4. Have you ever had severe headache attacks accompanied by hypersensitivity to lights and sounds?<br>Never<br>1–4 times<br>≥5 times<br>Unknown
Question 5. Have you ever had visual disturbances lasting 5–60 min followed by headache?<br>Never<br>1–2 times<br>≥3 times<br>Unknown

a Screener versus final ICHD-II diagnosis of migraine as gold standard

Screener question	Answer to screener question	Final diagnosis		Test characteristics (95% CI)
		migraine	no migraine	
(1) Self-reported migraine	Yes, ever (n)<br>No, never (n)	43<br>5<br>Sens 0.90 (0.77–0.97)	19<br>149<br>Spec 0.89 (0.84–0.94)	PPV 0.69 (0.57–0.82)<br>NPV 0.97 (0.93–0.99)
(2) Diagnosed with migraine by a physician	Yes, ever (n)<br>No, never (n)	26<br>20<br>Sens 0.57 (0.41–0.71)	8<br>160<br>Spec 0.95 (0.91–0.98)	PPV 0.76 (0.59–0.89)<br>NPV 0.89 (0.84–0.94)
(3) Severe headache with nausea	Yes, ever (n)<br>No, never (n)	42<br>5<br>Sens 0.89 (0.77–0.97)	17<br>148<br>Spec 0.90 (0.85–0.94)	PPV 0.71 (0.58–0.82)<br>NPV 0.97 (0.93–0.99)
(4) Severe headache accompanied by hypersensitivity to light and sound	Yes, ever (n)<br>No, never (n)	44<br>2<br>Sens 0.96 (0.85–1.00)	18<br>149<br>Spec 0.89 (0.85–0.94)	PPV 0.71 (0.58–0.82)<br>NPV 0.99 (0.95–1.00)
(5) Visual disturbances followed by headache	Yes, ever (n)<br>No, never (n)	28<br>15<br>Sens 0.65 (0.49–0.79)	20<br>144<br>Spec 0.88 (0.83–0.93)	PPV 0.58 (0.43–0.72)<br>NPV 0.91 (0.85–0.95)
All questions combined	Yes, ever (n)<br>No, never (n)	20<br>23<br>Sens 0.47 (0.31–0.62)	5<br>156<br>Spec 0.97 (0.93–0.99)	PPV 0.80 (0.59–0.93)<br>NPV 0.87 (0.82–0.92)

Sens = Sensitivity; Spec = specificity; missing data <5%.

b Visual disturbances followed by headache versus final ICHD-II diagnosis of migraine with aura as gold standard

Screener question	Answer to screener question	Final diagnosis		Test characteristics (95% CI)
		migraine with aura	no migraine with aura	
(5) Visual disturbances followed by headache	Yes, ever (n)<br>No, never (n)	18<br>2<br>Sens 0.90 (0.68–0.99)	30<br>157<br>Spec 0.90 (0.68–0.99)	PPV 0.38 (0.24–0.53)<br>NPV 0.99 (0.96–1.00)

Sens = Sensitivity; Spec = specificity; missing data <7%.

1. van der Willik D, Pelzer N, Algra A, Terwindt GM, Wermer MJH. Assessment of Migraine History in Patients with a Transient Ischemic Attack or Stroke; Validation of a Migraine Screener for Stroke. *Eur Neurol*. 2017;77(1-2):16-22. doi:10.1159/000449425

In the present study, migraine diagnosis was considered positive when participants answered confirmatively to a combination of the three questions selected to obtain the highest possible predictive value (ever migraine attack, ever severe headache with nausea/vomiting or ever severe headache with photo- and/or photophobia, positive predictive value: 0.80 (0.66-0.90) negative predictive value: 0.96 (0.91-0.98))[5] or in case of migraine diagnosis by physician's report.
